# Genome Sequence of Mycobacteriophage Lilbit

**DOI:** 10.17912/micropub.biology.001875

**Published:** 2026-09-15

**Authors:** Ellie Clavette, Andrea Chuva, Nicholas Gruener, Juniper Zinck, Sharon Gusky

**Affiliations:** 1 STEM, Connecticut State Community College Northwestern, Winsted, CT USA; 2 Torrington High School, Torrington, CT USA

## Abstract

Mycobacteriophage Lilbit was isolated using *Mycobacterium smegmatis* mc²155. It has a genome consisting of 65,106 base pairs with 110 putative genes and GC content of 63.4%. Based on gene content similarity, it is assigned to actinobacteriophage cluster S. Functions were predicted for 40 genes. There were no tRNAs identified in the genome.

**Figure 1. Plaques and Virion Morphology for Lilbit:** Lilbit produces plaques with halos that are ~ 1 mm in diameter (A) and is composed of a capsid 69.81 +/- 3.13 nm in width with a tail 283.58 +/- 7.02 nm in length (B):note the dark specs are artifacts.

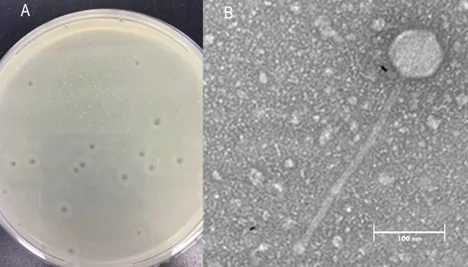



## Description

Bacteriophages are increasingly being studied for their potential use as therapeutic agents. Mycobacteriophages have been used to treat antibiotic-resistant infections in patients with cystic fibrosis, where *Mycobacterium abscessus* infection can be detrimental to the lungs, as well as in cases of infections by *Mycobacterium avium* and *Mycobacterium chelonae.* (Nick et al. 2022, Dedrick et al. 2023). Here we report on the isolation and characterization of a novel mycobacteriophage, Lilbit, which was isolated using *Mycobacterium smegmatis* mc ^2^ 155 as a host. There are a number of disease-causing organisms in the Mycobacterium genus including multi-drug resistant strains of *M. tuberculosis* and *M. leprae* which cause tuberculosis and leprosy, respectively. (Garg etal., 2017) Some bacteriophages isolated using *Mycobacterium smegmatis* will be able to infect other members of the genus. (Poxleitner 2018)

Lilbit was isolated from a sample of composting plant material collected in New Haven, Connecticut (GPS coordinates: 41.335200, -72.941760) using an enriched isolation method. The sample was resuspended in 7H9 liquid medium and inoculated with *M. smegmatis* mc ^2^ 155. After incubation with shaking at 42˚C for several days, the culture was filtered and the filtrate plated in top agar with *M. smegmatis* , resulting in plaques of phage Lilbit, which were purified through multiple rounds of plating until consistent tiny plaques measuring 1mm in size were obtained. Negative stain transmission electron microscopy using uranyl acetate stain revealed Lilbit to possess siphovirus morphology characterized by a non-contractile and flexible tail.

Lilbit's DNA was extracted using the Promega Wizard DNA kit and sequenced by the Pittsburgh Bacteriophage Institute using an Illumina MiSeq (v3 reagents), with libraries prepped using the NEB Ultra II FS kit. This produced 195,422 single-end 150 base raw reads that were assembled using Newbler version 2.9 into a 65,106 base-pair genome with a shot-gun coverage of 419. The assembly and genome termini was checked using Consed V29 (Gordon and Green, 1988), revealing an 11bp 3’ single-strand overhang of 5’-GCGCGCAGCGC at the termini. Lilbit was assigned to cluster S based on having a gene content similarity of at least 35% to the phages already assigned to cluster S. (Pope et al., 2017). Like other cluster S phages Lilbit has putative HNH endonucleases, putative methyltransferase, and multiple putative glycosyltransferases. (Sevcik et al., 2023)

The sequenced genome was auto-annotated using the Phage Evidence Collection And Annotation Network (PECAAN) v20221109 (Rinehart et al., 2015) using Glimmer v3.02 (Delcher et al., 2007) and GeneMark v4.28 (Lukashin and Borodovsky 1998), with start sites manually refined with Phamerator v606 using Actino draft database v578 (Cresawn etal., 2011) and Starterator v1.2 (Pacey 2016). BLAST, using the Actinobacteriophage BLAST program (Russell and Hatfull 2017) and NCBI non-redundant database v2.2.18 (Altschul et al., 1997) and HHPRED, using the PDB_mmCIF70, Pfam- v36, NCBI Conserved Domains databases (Söding 2005), and NCBIs Conserved Domains databases (Geer et al., 2015), were used to predict the gene functions. DeepTMHMM v1.0 (Chaturvedi 2011) was used to determine if any putative genes coded for transmembrane proteins. Aragorn v1.2.38 (Laslett and Canback 2004) and tRNA scan SE v2.0 (Chan et al., 2021) were used to check for the presence of tRNAs. Default settings were used for all software.

Lilbit was found to encode a total of 110 putative genes, 40 for which putative functions could be assigned. There were 96 genes transcribed in the forward direction, and 14 transcribed in the reverse direction. No tRNAs were found. A programmed -1 translational frameshift was identified for the genes which are predicted to code for tail assembly chaperones. These occur just before a 5,550 bp long gene predicted to encode the tape measure protein. Mycobacteriophage Lilbit is predicted to be lytic based on the absence of identifiable integrase or immunity repressor functions, consistent with other cluster S phages.

**Nucleotide sequence accession numbers**

Mycobacteriophage Lilbit is available at GenBank with Accession No. PV876982 and Sequence Read Archive (SRA) No. SRX29714289 .
